# Fluoroquinolone Resistance in Campylobacter Absent from Isolates, Australia

**DOI:** 10.3201/eid0911.030336

**Published:** 2003-11

**Authors:** Leanne Unicomb, John Ferguson, Thomas V Riley, Peter Collignon

**Affiliations:** * OzFoodNet, Newcastle, Australia; † University of Newcastle, Newcastle, Australia; ‡ Western Australian Centre for Pathology & Medical Research, Perth, Australia; § The Canberra Hospital, Garran, Australia

## Abstract

Fluoroquinolone resistance was detected in 12 of 370 Australian human Campylobacter isolates; 10 of these were travel-associated, and for 2 isolates travel status was unknown. No resistance was found in isolates known to be locally acquired. In Australia, fluoroquinolones have not been licensed for use in food production animals, a policy that may have relevance for countries with fluoroquinolone-resistant Campylobacter.

In Australia, Campylobacter is the most commonly reported bacterial foodborne pathogen with an annual incidence of 125/100,000 population (*1*). Fluoroquinolone resistance in this pathogen is recognized as an emerging public health problem related to the use of these antimicrobial agents in food production animals. Data from many regions (United States, Europe, and Thailand) that have licensed fluoroquinolones for therapeutic use in animals have shown that such use results in the emergence of fluoroquinolone resistance in *Campylobacter jejuni* and *C. coli* isolates obtained from both humans and animals (*2*–*4*). Increasing resistance in Campylobacter may lead to infections that are unresponsive to antimicrobial drug treatment and more severe disease. Smith (*2*) demonstrated that resistant *C. jejuni* caused more prolonged diarrhea in patients than susceptible strains.

Fluoroquinolone resistance may emerge during treatment in humans (*3*); however, Smith (*2*) and others (*5*) demonstrated that most detected resistant isolates come from patients who have not been exposed to fluoroquinolones. Furthermore, as human-to-human transmission of Campylobacter is rare, patients infected with resistant Campylobacter are not an important source of resistance for other humans (*4*).

In Australia, fluoroquinolones have never been licensed for use in food production animals. A small amount is used in companion animals: imports of enrofloxacin began in 1995, and 49 kg was used in the financial year 1996–97 (*6*). In contrast, the average use of quinolones in humans was 3,200 kg per year from 1992 to 1997 (*6*).

Australian data on fluoroquinolone resistance in human Campylobacter isolates are limited. As part of a case-control study of risk factors for Campylobacter infection conducted in New South Wales from 1999 to 2001, patients infected with this pathogen were recruited, and information on various exposures was obtained by telephone interview. Patients were asked about local and international travel in the 4 weeks before onset of diarrhea. Isolates from patients were stored and subsequently tested for resistance to 10 antimicrobial agents by using the National Committee for Clinical Laboratory Standards method for *Helicobacter* species (*7*). The Table shows proportions of fluoroquinolone-resistant isolates from this case-control study. In addition, results of two laboratory-based surveys of antimicrobial resistance, one conducted on isolates from Western Australia and one conducted on isolates from the Australian Capital Territory, are included. In these last two studies, information on overseas travel was sought retrospectively. Fluoroquinolone-resistant human Campylobacter isolates were rarely detected in Australia. All ciprofloxacin-resistant isolates detected in the three regions were from patients who appeared to have acquired their infection outside the country (Table). Two locally acquired isolates in the New South Wales study were resistant to nalidixic acid only (i.e., they were sensitive to fluoroquinolones).

**Table:** Fluoroquinolone resistance data for Australian Campylobacter isolates

Study location	Isolate source and collection period	Total no. tested	Proportion^a^ of fluoroquinolone-resistant isolates (%)			
			Locally acquired	Overseas acquired	Unknown acquisition status	
New South Wales	Human feces 1999–2001	180^b^	0/144 (0)	3^c^/7 (43)	2^c^/29 (6.9)	
Western Australia	Human feces 1999–2000	50^b^		4^c^	0/46 (0)	
Australian Capital Territory	Human feces/blood 2001–2002	140^d^		3	0/137 (0)	

^a^No. of resistant isolates by acquisition status/total no. isolates tested in acquisition status category. ^b^Testing by agar dilution, Mueller-Hinton agar with 5% lysed sheep blood (*7*). ^c^Resistant to ciprofloxacin (MIC > 4 mg/L). ^d^Testing by disc-susceptibility method (*8*).^.^

As Campylobacter infection is zoonotic, the absence of human, locally acquired infections attributable to fluoroquinolone-resistant organisms most likely reflects 1) the lack of use of fluoroquinolones in Australian poultry (the most common source for *C. jejuni*) and other potential meat sources and 2) the presence of little or no viable Campylobacter organisms on imported chicken, which has been a source of resistant Campylobacter infections in the United Kingdom (*5*). Only cooked chicken products can be imported into Australia.

Fluoroquinolones are critical therapeutic agents for many serious bacterial infections because, in many cases, they may be the only active oral agents available. Resistance following fluoroquinolone use can develop in many gram-negative bacteria (Campylobacter*,* salmonellae, and *Escherichia coli*) carried by animals. These bacteria can be present in food. If they subsequently cause infections in humans (or transfer their resistance genes to other bacteria), no effective antimicrobial agents may be available for treatment when serious disease occurs. Thus, their use in animals should be avoided. Australia has never licensed the use of fluoroquinolone agents in livestock. In contrast with other nations that have licensed their use, fluoroquinolone resistance in Campylobacter isolates and subsequent infections in humans acquired from meats eaten within the country have not emerged in Australia. The Australian experience has implications for the continued licensing of these agents in other countries in food production animals.

Antimicrobial testing of isolates from the New South Wales case-control study was funded by OzFoodNet, enhanced surveillance program of the Department of Health and Ageing, Australia.
